# Micromechanism Study of Molecular Compatibility of PVDF/PEI Blend Membrane

**DOI:** 10.3390/membranes12080809

**Published:** 2022-08-21

**Authors:** Ming Gao, Yuanlu Zhu, Jiangyi Yan, Weixing Wu, Beifu Wang

**Affiliations:** 1 College of Naval Architecture and Shipping, Zhejiang Ocean University, Zhoushan 316000, China; 2 College of Petrochemical Engineering and Environment, Zhejiang Ocean University, Zhoushan 316000, China

**Keywords:** polyvinylidene fluoride, polyetherimide, fluorine-amine bonds, blending, compatibility

## Abstract

In this paper, the compatibility of polyetherimide (PEI) with different contents as a high-performance copolymer and polyvinylidene fluoride (PVDF) was studied, and 5%–20% PEI was prepared by the non-solvent-induced phase inversion method. The compatibility of PVDF and PEI was evaluated by analyzing the physical structure and properties of the blend membrane, the microstructure, the glass transition temperature Tg, the enthalpy, and the mechanism of the polymer blend enthalpy change. The results show that the blend membranes have -NH and C=O-N binding energies at X-ray photoelectron spectroscopy (XPS), which preliminarily proves that fluorine–amine bonds are formed between the polymers, and new spectra appeared by Fourier transform infrared (FTIR) and X-ray diffraction (XRD) peaks, which further proves that the two have the formation of fluorine–amine bonds, the Tg and enthalpy of the mixed membrane was increased, and a scanning electron microscope (SEM) observed that the membrane pores changed from finger-like pores to sponge-like macropores. When the content of PEI is 15%, the performance of the blended membrane is the best, the water contact angle increases to 58.5°, the porosity increases to 17.33%, the maximum force increases to 8.04 N, and the elongation at break decreases to 24.26%, the pure water flux is 1870.292 L/m^2^·h, and the oil rejection is 87%. In addition, the enthalpy change of polymer blending further proves that PEI and PVDF are compatible systems and have a good performance improvement for PVDF.

## 1. Introduction

With the increasing requirements of membrane materials and membrane properties in the field of membrane technology, traditional membrane materials have been unable to meet the needs of technological development. The blending film complements the existing variety of membrane materials; sets a variety of membrane material properties in one; overcomes the shortcomings of single film materials; creates membrane materials with their own characteristics and complementary advantages; and provides more diverse choices in practical applications. From the various modification methods available in the literature, blending using polymeric materials that have targeted properties, is a simple and effective choice [1,2]. In recent years, mixed matrix membranes (MMMs) have gained much attention because of their advantages, which can provide many functions as multifunctional membranes. Leaper et al. [3] prepared MMMs for the purification of artificial sea water by incorporating graphene oxide functionalized with 3-(aminopropyl)triethoxysilane (APTS) into PVDF polymer solutions; compared with the original membrane, the permeability increased by 86% and the desalting rate was >99.9%. The blending particles distributed homogeneously and changed the overall properties of the membrane [4,5]. The quality and quantity of the blending are determined by several factors such as the composition of the dope solution, the temperature, the type of polymer material, and also solvents and additives [6,7].

Poly(vinylidene fluoride) (PVDF), one of the most widely used membrane-forming polymers, is a semi-crystalline polymer with a good membrane-forming performance, thermal stability, chemical stability, and mechanical properties [8,9]. This makes PVDF available in separation applications, namely microfiltration; Zhu et al. [10] designed a zterztant nano-hydrogel grafted poly(vinylidene fluoride) (PVDF) microfiltration membrane (ZNG-G-PVDF), which has excellent stain resistance and can be used for oil-in-water emulsion separation and ultrafiltration; and Cheng et al. [11] designed novel antifouling and anti-bacterial polyvinylidene fluoride (PVDF) ultrafiltration membranes with enhanced permselectivity, facilely fabricated through the one-step co-deposition of dopamine and a newly synthesized micromolecular zwitterion (DMAPAPS). Results showed that the strategy endowed the optimized membrane with a high pure water flux of 364 L/m^2^∙h and a BSA rejection of 98.6%. Prospective applications include drug delivery systems, water softening, biological separation, and sensory and protective coatings. However, *t* low wettability by polar compounds due to the hydrophobic surface, low surface energy, and lower heat resistance due to their lower melting point begins to limit the application of PVDF membranes to specific processes involving thermal and mechanical capabilities in the industry, and the surface strength is weak, which leads to the need to clean frequently in the process of use. Salem et al. [5], used a novel poly(ethersulfone)/polyvinylidene fluoride (PES/PVDF) blend membrane and its composite with modified graphene nanoplatelets (GNPs), and fabricated using the electro spinning technique. The compatibility between the two is good, but PES material also has the characteristic of being not easy to clean, so adding other polymer materials changes its performance, to promote the industrial application process of PVDF.

Poly(ether imide) (PEI) is also an amorphous high-performance polymer with excellent heat resistance, excellent dimensional stability, and a high reactivity. Its amide group can form fluorine bonds by covalent polymerization with fluorine–ammonia bonds [12]. The polymer poly(ether imide) (PEI) was blended into PVDF to form a blend membrane. PEI is a positively charged ion material and mixing with PVDF can enhance the interaction force between molecular chains and improve the performance of the membrane. The amide group in PEI can effectively improve the hydrophilicity of the membrane [13,14,15].

In this paper, PVDF/PEI blends were first prepared by the phase transformation method, and then the compatibility of PVDF/PEI blends was studied by FTIR, XRD, and XPS from the elemental structure, and further verified by DSC. Then, the morphology of the blend membranes with different additives was observed, the influence of different additives on the pore size structure and formation mechanism were analyzed, oil–water filtration experiments were carried out on blended films with different proportions, and the characterization results were analyzed to find out the optimal casting liquid ratio. In addition, the compatibility mechanism of the membrane was described by a systematic analysis of the blending system.

## 2. Experimental

### 2.1. Materials

Poly(vinylidene fluoride) (PVDF, A.R, M_W_ = 534,000) was provided by American manufacturer Aldrich Co., Ltd., Shanghai, China; poly(ether imide) (PEI, A.R, M_N_ = 25,000) was provided by American manufacturer SABIC Co., Ltd., Riyadh, Saudi Arabia; N,N-dimethylacetamide (C4H9NO, DMAc, A.R) was purchased from National Pharmaceutical Chemicals Co., Ltd., Beijing, China; and polyvinylpyrrolidone (PVP, A.R) was purchased from Yatai United Chemical Technology Co., Ltd., Suzhou, China.

### 2.2. Preparation of Blend Membrane

PVDF, PVP, and PEI were dried in a vacuum drying oven for 24 h. The materials were mixed in a certain proportion (Table 1) and added to the high-pressure reaction kettle and stirred at 60 °C for 24 h. The temperature was kept at 60 °C for 2 h, and the casting liquid was injected into the spinneret through gas. Deionized water was used as the core liquid, and the core liquid entered the spinneret through a peristaltic pump. The spinneret is a single hole spinneret (Φ 42*2.0*0.7), after the spinneret is extruded, it is solidified in the water bath and soaked in the water bath, so that the remaining solvent is evenly diffused, taken out, and dried for subsequent characterization experiments.

### 2.3. Membrane Characterization

#### 2.3.1. Basic Physicochemical Characterization

X-ray photoelectron spectroscopy (XPS Thermo Scientific K-Alpha, Waltham, MA, USA) is used to study the surface chemical composition of membranes. The spectral range was 0~1300 eV, and C1s peak was detected by a high-resolution spectrum. XPS full-scan spectral recording ranges from 0 to 1300 eV, with transmission energy of 150 eV, and a monochromatic Al Kα X-ray source of 1486.6 eV. FTIR-ATR was measured by Thermo Scientific Nicolet iS20 Fourier transform infrared spectrometer. The sample was placed on the sample holder and all spectra from 4000 cm^−1^ to 600 cm^−1^ were recorded. Interventionary studies involving animals or humans, and other studies that require ethical approval, must list the authority that provided approval and the corresponding ethical approval code.

#### 2.3.2. Crystalline Structure

The crystal structure of blend membranes was determined by a wide-angle X-ray diffractometer (WAXD, DX-2700, Shanghai, China). The scanning parameters include light source intensity (40 kV/30 mA), *λ* (1.54 A, Cu Kα line), light source slit width (0.6 mm), increment (1.20 °/min), and scanning range (5–80°). The crystal size of PVDF and PEI in the membrane is estimated by the Scherrer equation:

(1) $$D=\frac{K\lambda }{\beta \cos\theta }$$

where *D* is the estimated diameter (nm) of the crystal; *K* is Scheele’s constant (*K* = 0.89); and *λ* is the wavelength (nm) of incident X-ray, which is 0.154 in this study. β is the peak width (rad) at half height; and *θ* is the diffraction angle (rad).

The glass transition temperature of blend membranes in a nitrogen atmosphere was studied by a differential scanning calorimeter (DSC200-F3, NETZSCH, Germany). Approximately 8 mg of dry membrane is sealed in an aluminum pan. Hold at −80 °C for 3 min, then heat to 300 °C at a rate of 10 °C/min and held for 3 min to eliminate the heat history, then cool to 20 °C (10 °C min), recording the melting curve during this time. All tests were carried out in 0.05 mpa dry nitrogen atmosphere, with purge gas flow rate of 80 mL/min and guard gas flow rate of 30 mL/min.

#### 2.3.3. Membrane Morphology and Microstructure

The cross-sections and surface morphology of various membranes were observed under an accelerated voltage of 15 kV using a scanning electron microscope (SEM Gemini 300, ZEISS, Jena, Germany). Scanning electron microscopy (SEM) was used to examine the morphology of the upper surface and cross-section of various membranes at an accelerated voltage of 15 kV. When characterizing the cross-section morphology, the sample is prepared through a frac membrane after being completely cooled in liquid nitrogen. All samples are Au/Pd sputtered and carefully treated to avoid contamination.

#### 2.3.4. Membrane Hydrophilicity

The hydrophilicity of the membrane was measured by water contact angle measurement (WCA). The WCA of the membrane was measured using an optical contact angle tester (OCA15Pro, Datphysics Instruments GmbH, Filderstadt, Germany) according to the solid drop method. The contact angle can be expressed as the angle between the sample surface and the calculated droplet shape function, and the projection of the droplet image is called the baseline. Briefly, deposit a water drop on the membrane surface and observe and record the value of the contact angle until the water drop does not change during the short measurement time. An average is obtained by taking three parallel measurements of each sample.

#### 2.3.5. Membrane Porosity

After measuring the dry weight of the membrane, soak it in n-butanol for 12 h to get wet, and then measure the wet weight of the membrane. The porosity of the membrane can be obtained by the following formula:

(2) $$\varepsilon =1-\frac{\frac{w_{1}}{\rho_{m}}}{\frac{w_{0}-w_{1}}{\rho_{d}}+\frac{w_{1}}{\rho_{m}}}$$

where *ε* is porosity (%), *w*_0_ is dry membrane mass (g), and *w*_1_ is membrane mass after absorption of n-butanol (g). *ρ_d_* and *ρ_m_* indicate the densities of n-butanol (g/cm^3^) and polymer (g/cm^3^), respectively.

#### 2.3.6. Mechanical Properties of Membrane

The microcomputer-controlled electronic universal testing machine (CMT8501, Shenzhen, China) was used to measure the stress–strain curve and the breaking strength and elongation at the break of the membrane. The size of the selected membrane is 60 mm × 10 mm, and the distance between the two clips is 30 mm. Each sample was tested three times to obtain average strength. Obtained from Equations (3) and (4) respectively:

(3) $$R=\frac{F}{S}$$

where *R*, *F*, and *S* are, respectively, the membrane breaking strength (MPa), membrane breaking tension (N), and membrane area after breaking (mm^2^).

(4) $$\varepsilon =\frac{L}{L_{o}}$$

where *ε*, *L*, and *L_o_* represent elongation at break (%), final length (mm), and initial length (mm), respectively.

#### 2.3.7. The Permeability, Anti-Fouling Performance for the Membrane

Pure water flux (PWF) was measured with an ultrafilter at 0.1 MPa pressure. The flux was equilibrated for the passage of the first 30 min of permeation, whilst the following 10 min of permeation was collected. Pure water flux was evaluated by the following:

(5) $$J=\frac{V}{A\cdot \Delta t}$$

where *J* is the pure water flux (L/m^2^·h), *V* is the volume of penetrated water (L), *A* is the effective area of the membrane (m^2^), and *∆t* is the recorded time (h). All experiments were conducted thrice to obtain the results presented in this study, which were an average value.

UV/visible spectroscopy (TU-1901, General analysis) was employed to measure the concentration of feed solution and permeation of the oil solution at a wavelength of 294 nm. The membranes were tested using feed oil solution with concentration of 100 ppm. Then, the filtered solution was obtained in the same way as for water flux testing. The membrane oil rejection was then determined based on Equation:

(6) $$R=\left(1-\frac{C_{p}}{C_{F}}\right)\times 100\%$$

where *R* is the oil rejection (%) and *C_p_* and *C_F_* are the concentration of the permeate (ppm) and the feed (ppm), respectively.

## 3. Results and Discussion

### 3.1. Effect of PEI Content on Microstructure of Membrane

**Figure 1:**
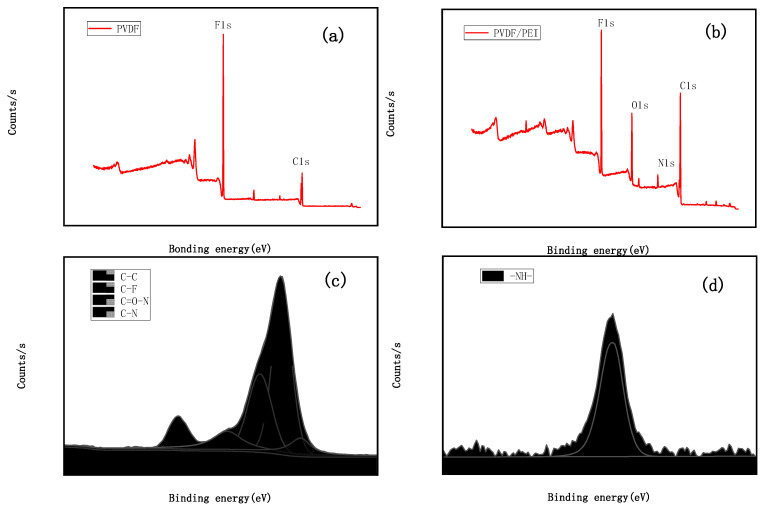
XPS spectrum of PVDF/PEI blend membrane. (**a**) Wide spectrum of PVDF pure membrane. (**b**) Wide spectrum of PVDF/PEI blend membrane. (**c**) C1s spectrum of PVDF/PEI blend membrane. (**d**) N1s spectrum of PVDF/PEI blend membrane.

Figure 1 shows the XPS spectrum after PEI blending, and Figure 1a shows the XPS spectrum of the PVDF pure membrane, which mainly contains two peaks, namely C1s with a binding energy of 284.8 eV and F1s with a binding energy of 687.2 eV. Figure 1b is the broad spectrum of the blend membrane after blending with PEI. Two new peaks appear, namely 407.5 eV N1s and 534.1 eV O1s. Table 2 shows that the content of N increases from 3.13% to 4.1%, and the content of O increases from 4.34% to 14.43%, indicating that PEI is compatible with PVDF. Compared with Figure 1c C1s spectrum of the PVDF pure membrane, a new peak of 287.5 eV C=O-N group appeared in the blend membrane [16]. The peak at 399.6 eV in the N1s spectrum in Figure 1d corresponds to the -NH- part, indicating that ring-opening occurred in PEI, which may be due to high temperature, and a fluorine bond was formed with PVDF through a covalent bond interaction, proving that the two are compatible systems.

**Figure 2:**
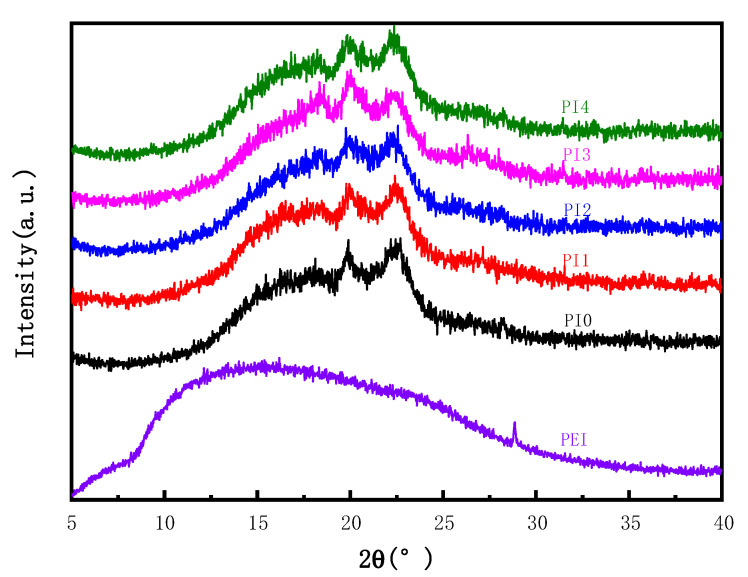
XRD patterns of blended membranes with different PEI contents.

Figure 2 shows the effect of PEI content on the XRD of hollow fiber membranes. It can be seen from the figure that PVDF showed typical α and β characteristic peaks (23.3° and 20.1°). With the increase in PEI content, the α crystal diffraction peak at approximately 23.3° changes gradually to the β crystal peak at 20.1°, presenting an amorphous hollow pattern. Since PEI is a kind of amorphous material, as shown in the XRD pattern, the PEI curve is a broad peak, which conforms to the XRD pattern of amorphous materials, the addition of PEI can make PVDF form a directional β crystal, forming a new hydrogen bond, namely the formation of a fluorine amine bond, enhancing the intermolecular force, which is consistent with the XPS experiment, thus promoting the increase in the β crystal characteristic peak. However, there is a wide diffraction peak between 15° and 18.6°, which is the characteristic peak of PEI, and the intensity gradually increases with the increase in content, which can prove that the compatibility of PEI and PVDF is further improved. The characteristic peak of PI4 began to decrease at the beginning of PEI, which may be due to the increase in the content of PEI with a positive charge. P-phenyl no longer acts as an electron acceptor, and the fluorine ion turns into an electrophilic ion, which reacts with p-phenyl as an electron acceptor, thus weakening the electrostatic force between molecules.

The FITR spectrum can be used to determine different crystal type contents in the blend, and different absorption bands correspond to PVDF polycrystalline substances. The peak heights (area) at 761 cm^−1^ (α type) and 839 cm^−1^ (β type) in the spectrum were picked. The formula was used to calculate the β-type content of the blend [17]:

(7) $$F\left(\beta \right)=\frac{A_{\beta }}{\left(1.26\right)A_{\alpha }+A_{\beta }}$$

*F(β)* is the content of the β crystal, and the height of the absorption band corresponding to 761 cm^−1^ and 839 cm^−1^ is the height of the absorption band corresponding to *A_α_* and *A_β_*.

As can be seen from Table 3, the addition of PEI increased the β content of the blend, and the addition of PEI promoted the nucleation and growth of PVDF, which was consistent with the experimental results of XRD.

**Figure 3:**
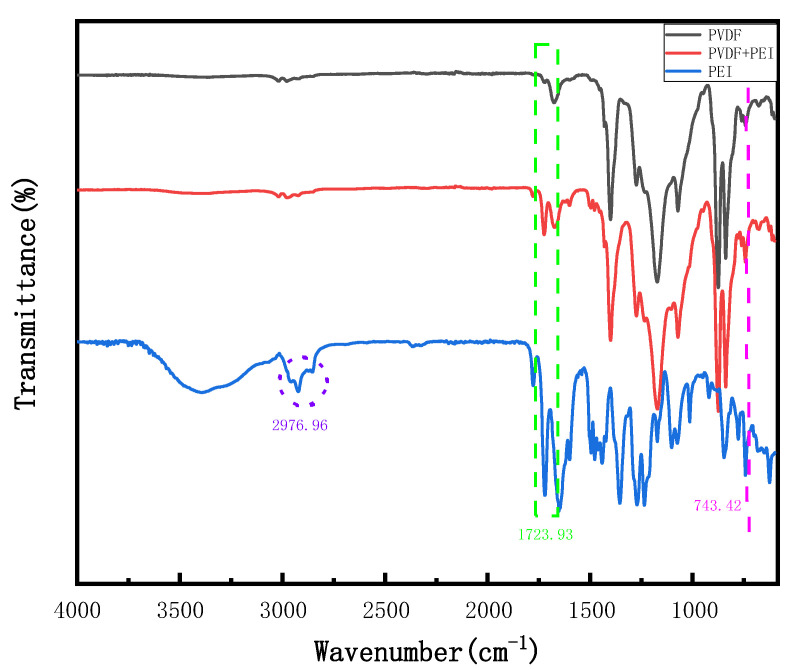
FTIR spectra of PVDF pure membrane, PEI pure membrane, and PVDF/PEI blend membrane.

As can be seen from the infrared spectrum in Figure 3, the peaks of 1071.72, 1173.24, 1274.35, 1400.50, and 1676.53 cm^−1^ are all formed by the C-F bond stretching oscillation of PVDF, and the emergence of the 700–900 cm^−1^ peak is related to the vibration of PVDF [18], 1000–1250 cm^−1^ and 1710–1720 cm^−1^ each attributed to the presence of C-O (C-O ether), C-N, and C=O functional groups, which were characteristic peaks of PEI, a semi-crystalline polymer. After the addition of PEI, the peak strength of 700–900 cm^−1^ decreased, which was due to the interaction between the polar groups of C-O ether bonds and the molecular chain structure of PVDF. The peak of 1676 cm^−1^ showed a band shift, which may be due to the ring-opening reaction of the PEI aromatic ring, the stretching oscillation of C=O-N, and the covalent bond interaction between amide bonds and PVDF, resulting in a new hydrogen bond [19]. The peak of 1723.93 cm^−1^ is due to the bending vibration of the -NH group in PEI, while the peak of 2976.96 cm^−1^ is due to the stretching vibration of -NH [16]. These peaks belong to the characteristic peak of PEI, which further indicates that PEI is successfully compatible with PVDF, which is consistent with the results of XRD.

### 3.2. Effect of PEI Content on DSC of Membranes

**Figure 4:**
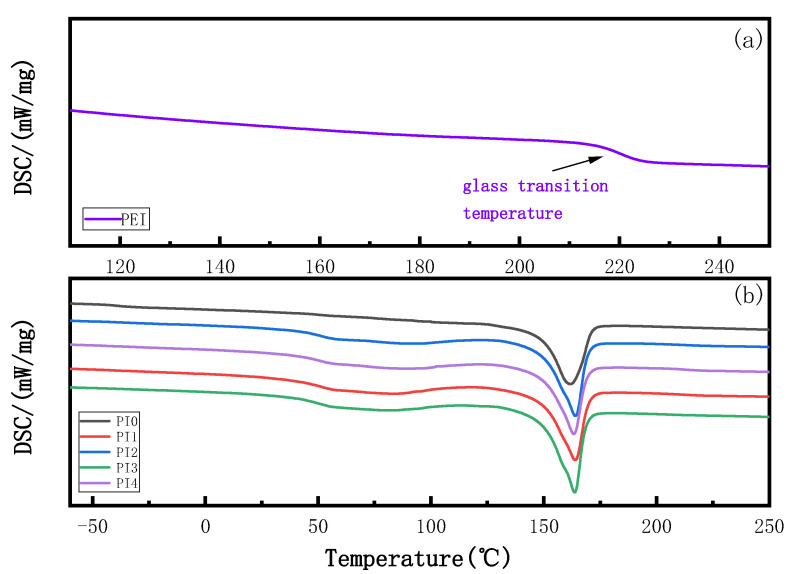
The kind of membrane DSC curves. (**a**) PEI pure membrane, (**b**) pure membrane and blend membrane curves.

Figure 4 shows the influence of PEI content on the glass transition temperature of the blend membrane. The DSC study shows that the glass transition temperature (Tg) of various blend membranes is shown in Table 4. The vitrification transition temperature of PVDF is −40.65 °C, which is consistent with that described in the literature [20], and the vitrification transition temperature of PEI in the relevant literature is 210–220 °C [21]; the glass transition temperature of PEI was 220.07 °C. After PVDF and PEI were blended, the glass transition temperature of the blend membrane increased significantly compared with that of the original membrane. However, with the increase in PEI content, the glass transition temperature of the blend membrane did not change much, which was consistent with the experimental conclusion. The melting temperature of the blended film did not change much compared with the pure film, but the enthalpy value was higher than that of the pure film, indicating that the crystallinity of the blended film increased. When the amount of PEI was 15%, the compatibility between PEI and PVDF was the best, and the addition of PEI promoted the nucleation and growth of PVDF, When the additive amount is 20%, the enthalpy decreases, the compatibility deteriorates, and the agglomeration of PEI may occur. Compared with the pure PVDF membrane temperature, Tg increased after PVDF and PEI blending, indicating that there is an interaction between PVDF and PEI. The amide group in PEI interacts with the fluorine bond in PVDF ; as a result, the spatial chain segments between polymers are destroyed, and the reduction of free volume limits the movement of molecular chains, thus forming a compact structure. Therefore, more heat is needed to activate the interchain movement of polymers, leading to the increase in Tg in the blend membrane.

### 3.3. Effect of PEI Content on Membrane Morphology

**Figure 5:**
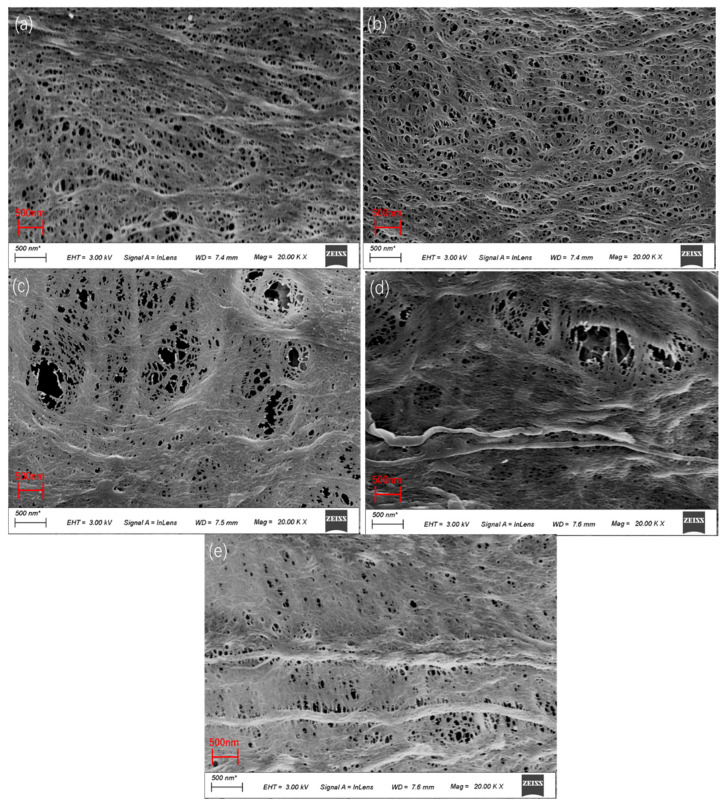
SEM surface morphology of PVDF/PEI blend membrane. (**a**) PI0, (**b**) PI1, (**c**) PI2, (**d**) PI3, (**e**) PI4.

Figure 5 shows the SURFACE SEM diagram of the PVDF/PEI blend membrane. From the surface morphology, it can be seen that all membranes present a symmetric structure, and the addition of PEI does not change the overall structure of the membrane. With the increase in PEI content, the membrane began to appear in a honeycomb structure, this is because PEI is a hydrophilic substance, the hydrophilic influence of amide groups on the surface indicates that PVDF and PEI have a good compatibility [22]. With the increase in PEI content, crude fiber exists at 20%, which may be because the electrostatic force between PEI and PVDF is weakened due to the increase in PEI content, which reduces the diffusion rate between the solvent and the non-solvent. Polymer precipitation is formed before the polymer-rich phase solidifies into a membrane, and the resulting PEI appears as an agglomeration phenomenon.

**Figure 6:**
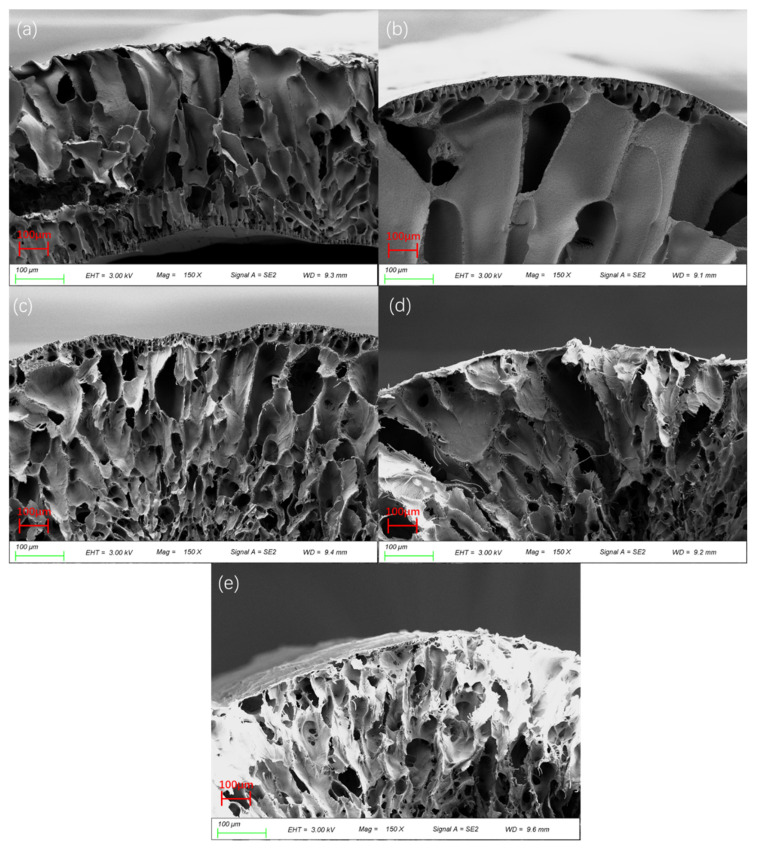
SEM cross-sectional morphologies of PVDF/PEI blend membrane. (**a**) PI0. (**b**) PI1. (**c**) PI2. (**d**) PI3. (**e**) PI4.

Figure 6 shows the SEM diagram of the cross-section of the PVDF/PEI blend membrane. The pore structure of the pure PVDF membrane mainly presents a typical finger-like pore structure. With the increase in PEI content, the number of finger-like pores gradually decreases while the number of spongy pores gradually increases. The addition of PEI molecules speeds up the transient phase in the membrane-forming process, and the diffusion rate of the solvent and the non-solvent is increased, which makes it easy to form macroporous structures [19]. When the PEI content increased to 20%, the direct connectivity between the channels became poor, because PEI agglomeration increased the instability of the whole system in the dynamics, and the interchain forces of PEI inhibited PVDF nucleation and growth.

### 3.4. Effect of PEI Content on Membrane Water Contact Angle

**Figure 7:**
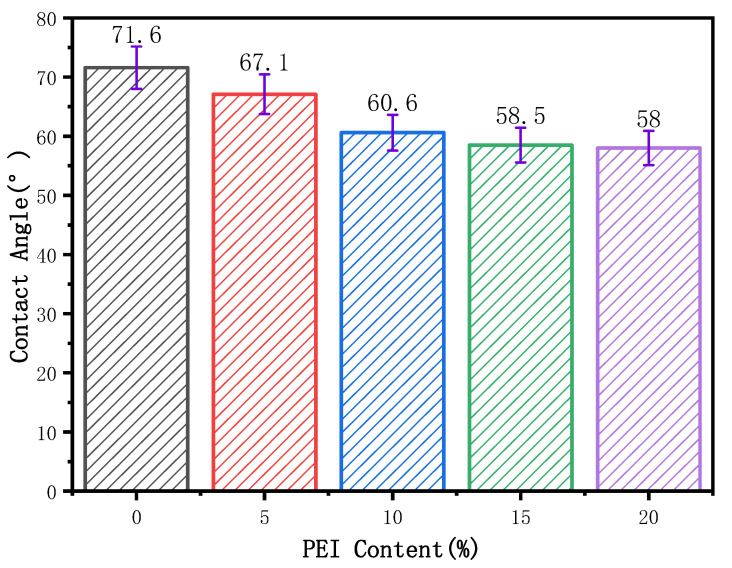
Effect of PEI content on contact angle of blended membrane.

Figure 7 shows the influence of the PEI content on the hydrophilicity of the blend membrane. As can be seen from the figure, with the increase in PEI content, the contact angle of the membrane presents a downward trend, from 71.6 in PI0 to 58° in PI4. PI0 is the pure PVDF membrane, and PVDF itself has excellent hydrophobicity, so the degree of the water contact angle is high. When the PEI content began to increase, the water contact angle began to decrease gradually, this is because PEI is a hydrophilic material, its surface with amino groups and polar bonds and ether bonds are hydrophilic groups, so that the surface of the blend membrane’s hydrophilicity increases. In addition, the increase in spongy pores also leads to an increase in hydrophilicity, which is consistent with the results of SEM. However, the water contact angle of PI3 and PI4 is not very different, which may be due to the agglomeration of PEI that makes the connectivity of the surface layer worse and the hydrophilic groups no longer increase. When the PEI mass fraction was 15%, the water contact angle was 58.5°, which was 15.6% lower than that of the PVDF pure membrane.

### 3.5. Effect of PEI Content on Membrane Porosity

Table 5 shows the influence of PRI content on the porosity of the hollow fiber membrane. It can be seen from the table that the porosity firstly increased and then decreased. In PI0, 7.35% increased to 17.33% of PI3 [22]. This was because the instantaneous phase separation led to the formation of cavernous pores with a large pore size, which increased the pore structure inside the membrane, which was consistent with the SEM analysis results. The reason for the decrease in porosity is that due to the high content of PEI, the thermodynamic instability leads to the agglomeration of polymers, and the compatibility between PEI and PVDF decreases, thus the generation of crude fiber affects the pore size structure. When the mass fraction of PEI increased from 0% to 15%, the porosity increased from 7.35% to 17.33%, increasing by 135.8%.

### 3.6. Effect of PEI Content on Mechanical Properties of Membrane

**Figure 8:**
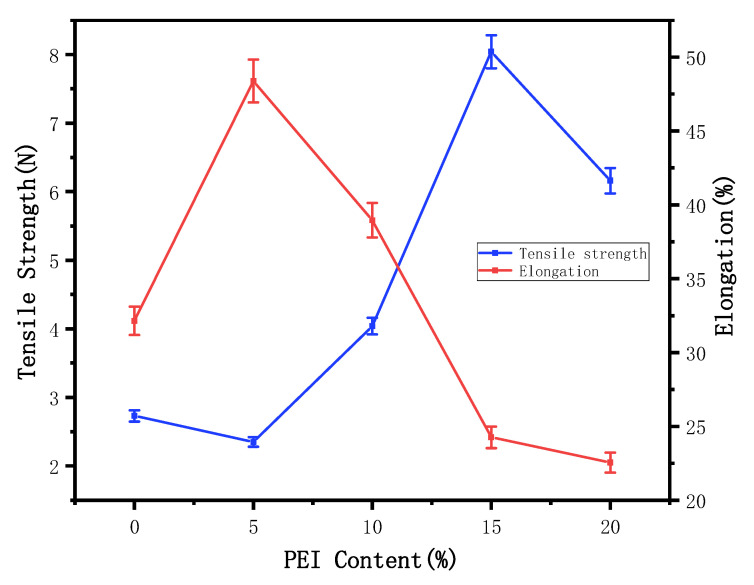
Effects of PEI content on tensile strength and elongation of membrane.

Figure 8 shows the influence of PEI content on the maximum strength and elongation at the break of the membrane. Due to the general law of rigid materials, when the PEI content increases to a certain level, the overall structure will show an obvious improvement. At this time, the aromatic ring groups on PEI affect the main chain molecules of PVDF, the rotation degree of freedom of the primary chain is reduced, the distance between the chains is increased, the interaction between the chains is enhanced, and the rigidity of the membrane is improved. Along with the increase in the content of PEI in the elongation at break and the most strong overall showed a trend of decrease after the first increase, this is because the PVDF is a kind of vinyl semicrystalline material, so as the polar materials are added to a certain amount, the main chain rigidity reaches a certain degree, the interaction between the chain began to decrease, and the maximum force and elongation at break will decreased. When the additional amount of PEI reached 15%, the maximum strength increased from 2.73 N to 8.04 N, increasing 194.51%, and the elongation at break decreased from 32.15% to 24.26%, decreasing 32.52% [23].

### 3.7. Filtration Test of Membrane

**Figure 9:**
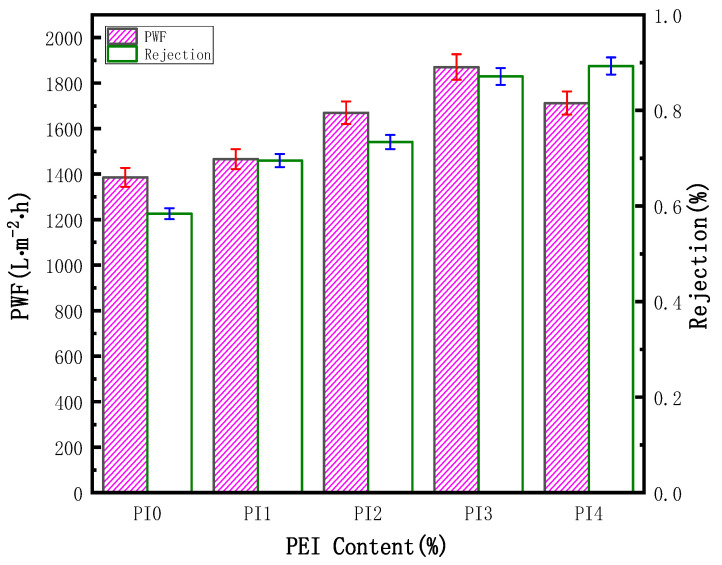
Pure water flux and oil rejection rates of various PVDF/PEI membranes.

Oil filtration experiments were performed to evaluate the separation and retention performance of the membranes. The results of permeability flux and oil rejection are shown in Figure 9. The permeability flux first increased, then decreased, while the trend of the rejection rate has been increasing. A small amount of PEI can help PVDF to form cavernous macropores and increase the porosity, so as to improve the permeability of the blend membrane. On the contrary, when the PEI reached a certain content, a relatively compact structure was formed on the membrane surface, which reduced the flux to a certain extent, which was consistent with the observation by SEM. The addition of the hydrophilic polymer PEI improved the hydrophilicity of PVDF and enhanced the oil separation performance. Compared with the PVDF hollow fiber membrane, the rejection rate of the PI3 blend membrane increased by 29%.

### 3.8. Compatibility Mechanism Analysis

According to the relation between ΔHm and the critical value (0.0419 J/mol when ΔGm = 0), the compatibility of the blend system is judged, that is, when ΔHm is less than the critical value, it is completely compatible, when ΔHm intersects with the critical value, it is partially compatible, and when δ Hm is greater than the critical value, it is incompatible. ΔHm is calculated according to Equation (8) [24]:

(8) $$\Delta H_{m}={\left\{X_{f}M_{f}\rho_{f}{\left(\delta_{f}-\delta_{i}\right)}^{2}{\left[\frac{M_{i}}{\left(1-X_{i}\right)M_{\dot{i}}\rho_{i}+\left(1-X_{f}\right)M_{f}\rho_{f}}\right]}^{2}\right\}}^{\frac{1}{2}}$$

where *X_f_* and *X_i_* are the mass fractions of PVDF and PEI, *M_f_* and *M_i_* are the molecular weights of PVDF and PEI, *ρ_f_* and *ρ_i_* are the densities of PVDF and PEI, and *δ_f_* and *δ_i_* are the solubility coefficients of PVDF and PEI.

**Figure 10:**
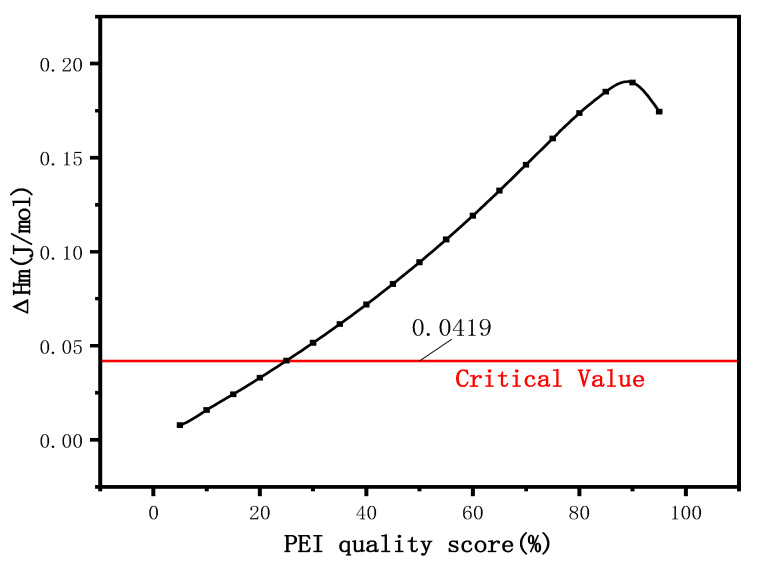
Enthalpy change of PVDF/PEI blend membrane.

**Figure 11:**
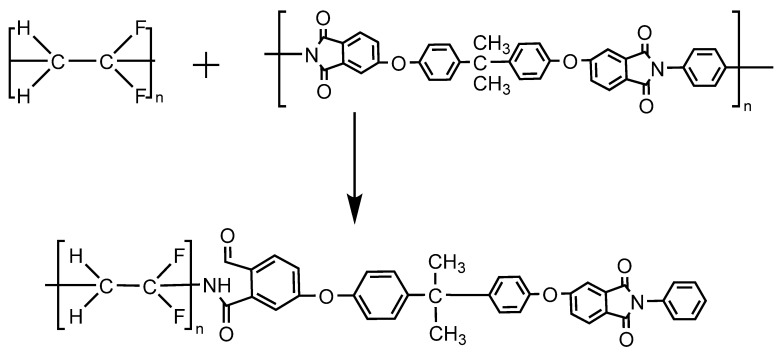
Mechanism of intermolecular chain reaction of PVDF/PEI blend membrane.

According to Figure 10, ΔHm intersects with the critical value. According to Schneier’s theory, the blending system is partially compatible. When the PEI content is below 25%, the blending system ΔHm is less than the critical value, and the theory can be seen as two kinds of polymer for compatibility, as shown in Figure 11. At this time of the PEI, the interaction between phenyl carries a positive charge and PVDF, the ring-opening reaction to form the key, the fluorine amine hydrogen bonding force between two kinds of polymer is enhanced, and the interaction force between the chain; and when the PEI content is more than 25%, because of the increasing concentration of PEI, and crosslinking each other between the molecules in the PEI no longer as the electron acceptor, phenyl into electrophilic fluorine ions as the electron acceptor and the phenyl crosslinking reaction, so the electrostatic force is reduced, the PEI begins to inhibit the nucleation of PVDF, at this time of Δ Hm is greater than the critical value. The two polymer systems are complementary compatible systems.

## 4. Conclusions

PVDF and PEI are blend systems with good compatibility. The -NH peak appeared in the N1s spectrum of XPS in the blend membrane, which preliminarily proved that the ring-opening reaction took place in PEI. The new PEI characteristic peak and the oscillation and shift of the -NH band appeared in the FTIR study. In XRD, the crystallization peak changes, and the β crystal peak gradually increases, which further proves that the ring-opening reaction of PEI takes place and the fluorine–ammonia bond is formed with PVDF, which verifies that PEI and PVDF are compatible systems. Through further study and an analysis of glass transition temperature, Tg was significantly increased compared with the pure film, and only one Tg appeared, and the increase in enthalpy resulted in the increase in crystallinity of the blended film, which further proved the compatibility between PEI and PVDF. It can be found from the study of the morphology of the blend membrane that with the increase in PEI content, the diffusion rate of the solvent and the non-solvent accelerates, and the membrane pore structure changes from a finger-like pore to a sponge large pore. When the PEI content reaches 20%, the agglomeration phenomenon occurs, the diffusion rate decreases, and the connectivity between the pores deteriorates. When the PEI content is 15%, the blends show the best performance, the maximum strength of the blend membrane is 8.04 N, the elongation at break is 24.26%, and the addition of the hydrophilic amino group reduces the water contact angle to 58.5°, and the porosity increases to 17.33%, the pure water flux is 1870.292 L/(m^2^·h), and the oil rejection is 87%, Finally, the dissolution enthalpy method of the polymer blend was used to analyze the mechanism of the blend system. When the amount of PEI was less than 20%, the fluorine amine bond was formed between the covalent bond PVDF and PEI, and a new hydrogen bond was formed between the molecules, and the interaction force increased. When the addition amount is greater than 20%, the electrostatic force decreases, which proves that the two polymers are partially compatible systems.

## Figures and Tables

**Table 1:** PVDF/PEI blend membrane material ratio.

Blend Membrane	PEI/PVDF (17 wt.%)	PVP (6 wt.%)	DMAC (77 wt.%)
PI0	0/100	6	77
PI1	5/95	6	77
PI2	10/90	6	77
PI3	15/85	6	77
PI4	20/80	6	77

**Table 2:** XPS element content.

Element Type	Element Content (%)	
	PVDF	PVDF/PEI
F	42.99	18.00
N	3.13	4.10
O	4.34	14.43
C	49.55	63.47

**Table 3:** Spectral parameters, beta-content, and pure PVDF and its blends.

PVDF Blends	F (β) (%)
PVDF	90.73
PVDF/PEI	93.62

**Table 4:** DSC data of blend membranes.

Blend Membrane	Glass Transition Temperature (Tg)			Melting Temperature (Tm)	Enthalpy (J/g)
	Onset	Inflect Point	Endset		
PI0	−46.82 °C	−40.65 °C	−35.06 °C	161.82 °C	38.66
PI1	41.39 °C	47.57 °C	54.02 °C	163.76 °C	42.77
PI2	42.22 °C	48.88 °C	55.70 °C	163.93 °C	43.54
PI3	42.99 °C	50.08 °C	57.18 °C	163.95 °C	46.25
PI4	42.04 °C	48.08 °C	55.36 °C	163.36 °C	37.90
PEI	212.64 °C	220.07 °C	227.11 °C	---	---

**Table 5:** Effect of PEI content on porosity.

PVDF/PEI Membrane with Different Components	PI0	PI1	PI2	PI3	PI4
**Porosity (%)**	7.35	13.47	16.41	17.33	6.56

## Data Availability

The data used to support the findings of this study are available from the corresponding author upon request.
